# Best practices in diagnosis and treatment of chronic iliac vein obstruction

**DOI:** 10.1590/1677-5449.190134

**Published:** 2020-09-14

**Authors:** Fabio Henrique Rossi, Thiago Osawa Rodrigues, Nilo Mitsuru Izukawa, Antônio Massamitsu Kambara

**Affiliations:** 1 Instituto Dante Pazzanese de Cardiologia de São Paulo – IDPC-SP, São Paulo, SP, Brasil.; 2 Centro de Intervenções Endovasculares – CIEV-IDPC, São Paulo, SP, Brasil.

**Keywords:** May-Thurner Syndrome, Cockett Syndrome, iliac vein, venous thrombosis, angioplasty, stenosis, stent

## Abstract

Iliac vein obstruction occurs in 20-30% of the general population. In patients with severe chronic venous insufficiency, this prevalence can be even higher, reaching 50-90% when the obstruction is investigated using intravascular ultrasound. Less invasive methods, such as venous Duplex Scanning, and even invasive ones such as venography may fail to diagnose the condition. Endovascular treatment of these obstructions is effective, safe, and associated with excellent clinical outcomes and stent patency rates, provided that fundamental anatomical and technical principles are considered and applied.

## INTRODUCTION

Iliac vein obstruction (IVO) can be classified as primary, non-thrombotic iliac vein obstruction (NTIVO), classically known as May-Thurner Syndrome (MTS); or secondary, postthrombotic iliac vein obstruction (PTIVO), or Cockett’s Syndrome (CS).^1^ Currently, endovascular treatment is considered the gold standard and it is associated with clinical improvement and improved quality of life, low morbidity and mortality, and high patency rates.^2^^-^^4^ In cases of venous insufficiency with clinical, etiological, anatomic, and pathophysiological classification (CEAP) grades C3 to C6, intravascular ultrasonography (IU) can detect iliac vein obstruction in 50 to 90% of investigated limbs.^2^^,^^4^^,^^5^ Although compression occurs most frequently at the point at which the right iliac artery and the left iliac vein cross, it is not uncommon in other segments. In our patients, and in those of other authors, 30% of the obstructions identified were observed at points other than those described classically.^6^^-^^8^ In addition to reduced cross-sectional area of the vessel, this compression can provoke formation of fibroblast membranes and adhesions, and also thrombosis^9^^-^^11^ (60% of obstructions in our patients were associated with a prior episode of deep venous thrombosis [DVT]).^4^ Phlebosclerosis (Rokitansky phenomenon), scarring retraction, and extensive obstructions are frequent in patients with DVT linked to compression of the femoral-iliocaval axis. It should be remembered that complete revascularization of the lumen of the affected vessel is only observed in 20 to 30% of cases.^11^ Other less common causes of compression include benign and malignant tumors, retroperitoneal fibrosis, iatrogenic injuries, radiation exposure, cysts, and aneurysms.

All of the clinical manifestations related to chronic venous insufficiency (CVI) can be caused by the obstructive injuries and the consequent venous hypertension. Refractory chronic pain, venous claudication, and edema are important predictive signs and symptoms of these obstructions.^1^^,^^9^^,^^12^^,^^13^ There is a positive association between severity of symptoms, clinical classification, and degree of IVO (p = 0.001).^6^ The combination of obstruction with venous reflux appears to be related to the more severe clinical cases.^13^^-^^15^ It has been observed that IVO can also be related to pelvic congestion syndrome,^16^ which was present in 26% of our patients.

## CLINICAL ASSESSMENT

At the initial consultation, the intensity of pain should be assessed using a visual analog pain scale (VAPS),^17^ CEAP classification should be assessed;^18^ diameters of limbs should be measured at the mid-thigh, leg, and mid-foot; the limb should be classified using the Venous Clinical Severity Score (VCSS);^19^ and the SF-36 quality of life questionnaire should be administered.^20^ It is important to determine severity and, most importantly, to observe patients’ clinical progress and response to treatment. In our clinical practice, for patients with CVI with CEAP ≥ C3, VAPS ≥ 3, VCSS ≥ 8, considerable impairment of quality of life, and ≥ 50% iliac obstruction, we offer endovascular treatment of the obstructed region.

## PATHOPHYSIOLOGY

A series of anatomic, physiological, and mechanical differences between the venous and arterial systems should be taken into consideration if treatment is to be successful. The venous system is convergent, with low velocity, high volume flow, and low pressure, in addition to having very high complacency. A small increase in venous capillary pressure can be responsible for the appearance of signs and symptoms. Decompression or deobstruction of the deep vein system and consequent reduction of ectasia and venous hypertension is the basic underlying principle of treatment. It should be remembered that the iliac vein is the principal route of drainage for venous flow from the lower limbs. However, even today, it is still not known at what degree or extension an obstruction becomes hemodynamically significant. Studies that analyzed direct pressure measurements proved inconclusive.^21^^,^^22^

## DIAGNOSTIC METHODS

**Figure 1:**
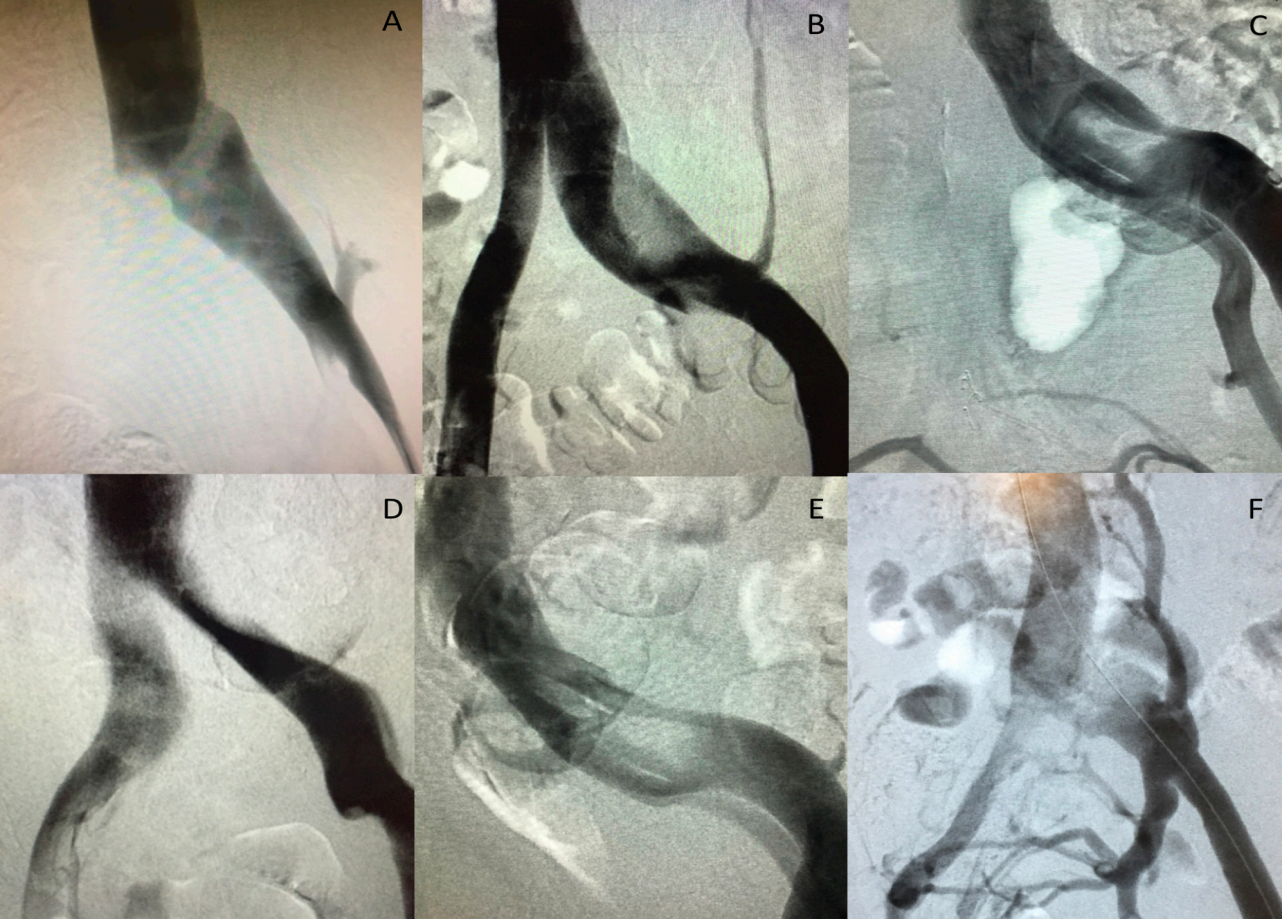
Digital subtraction phlebographic images of patients with iliac obstructions: (A) Pancaking and compression in a proximal segment of the left common iliac vein; (B) Severe compression of a proximal segment of the left common iliac vein and presence of a paravertebral collateral; (C) Presence of central rarefaction of contrast in a proximal segment of the left common iliac vein (bull's-eye sign); (D) Bilateral compression of the proximal common iliac veins; (E) Presence of intraluminal membranes in the left common iliac vein; (F) Severe obstruction of the left iliac vein with formation of a network of paravertebral and transpelvic collaterals.

Clinical experience demonstrates that treatment of obstructions ≥ 50% is related to improvement of symptoms and of quality of life and, therefore, it is considered that these obstructions are hemodynamically significant and should be treated.^4^^,^^14^^,^^23^ Since there is no reliable hemodynamic test, diagnosis and treatment are conducted on the basis of morphological analysis of obstructions. For a long time, venography was considered the gold-standard method and it can be a good diagnostic tool in cases with severe obstructions, but it fails when compared with intravascular ultrasound (IVUS) in at least 1/3 of cases.^4^^,^^24^ The indirect venographic signs that suggest the presence of IVO are: widening of the iliac vein (pancaking), central rarefaction of contrast (the bull's-eye sign), and presence of transpelvic or paravertebral collaterals (Figure 1).

Venous color Doppler ultrasonography is part of initial investigation of patients with CVI. It is a low-risk, noninvasive method. However, it is operator-dependent and even in specialized laboratories it can fail in up to 20% of cases.^7^^,^^25^ At our institution, comparison of direct and indirect measurements taken using this method against those taken with IVUS showed that a velocity ratio ≥ 2.5 across the point of greatest obstruction was the best parameter for diagnosis of ≥ 50% obstruction when compared with IVUS (r = 0.790; p < 0.001). That study proposed an algorithm that achieved 86.7% accuracy for ultrasonographic diagnosis of these obstructions (k = 0.73; p < 0.001).^7^

**Figure 2:**
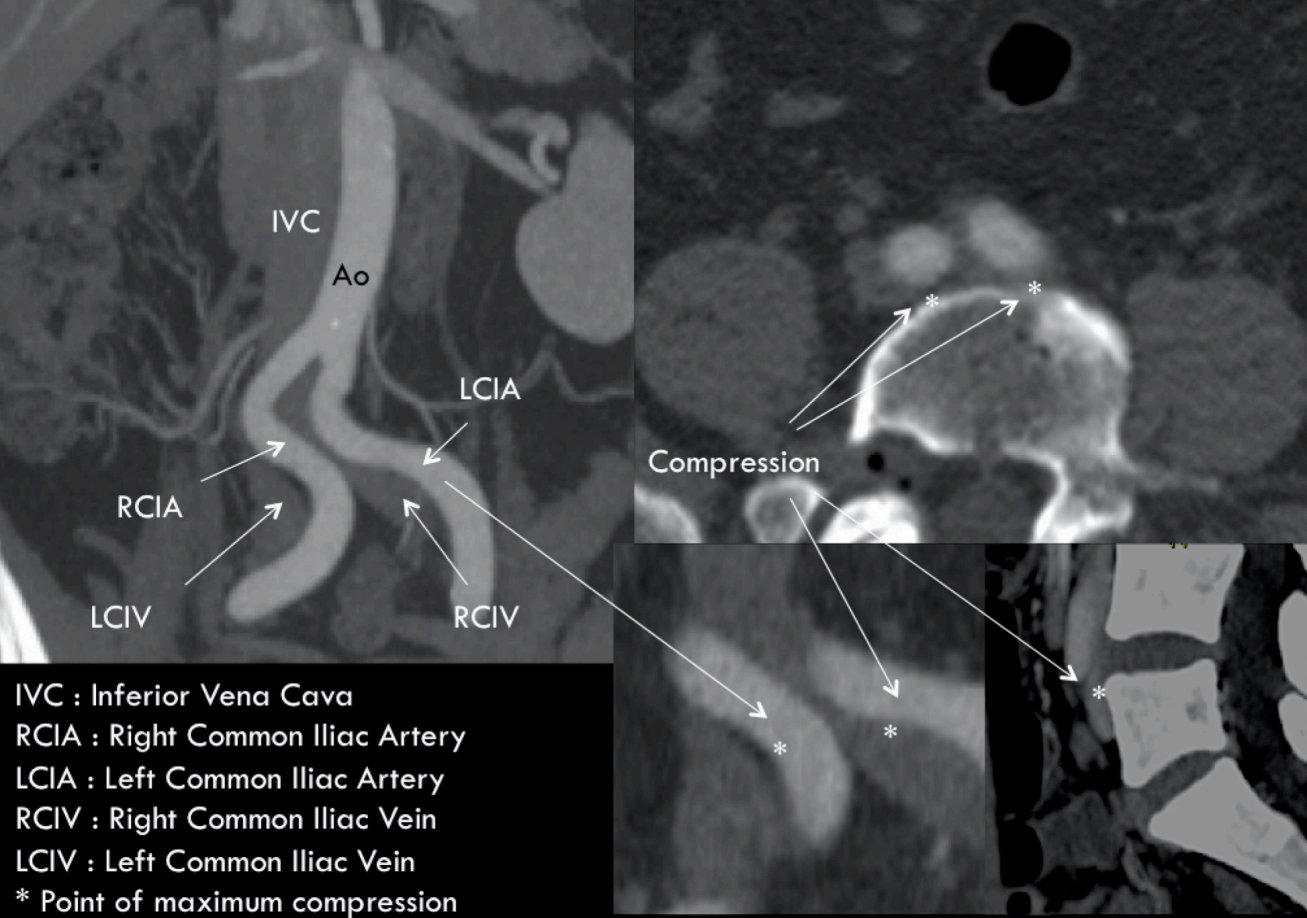
Multiplanar reconstruction of venous angiotomography to identify the point of maximum compression or obstruction.

**Figure 3:**
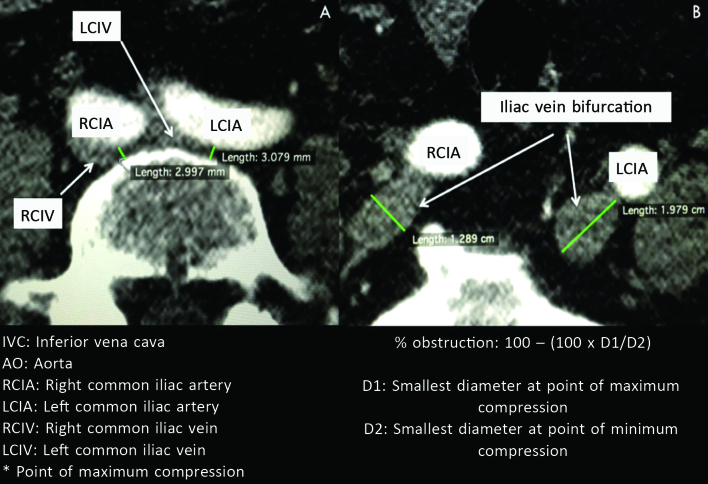
Calculation of the degree of obstruction at the point of maximum compression in limbs with chronic venous insufficiency with clinical, etiological, anatomic, and pathophysiological (CEAP) classification grades C3-6.

The utility of computed tomography (CT) for diagnosis of DVT has been widely described;^26^^-^^29^ but very few studies have tested its accuracy for identification of chronic IVO. Rossi et al. evaluated the diagnostic power of CT for determination of the degree of iliac obstruction by means of 3D multiplanar reformation of images, when compared with IVUS in patients with advanced CVI (CEAP C3-6), observing that in 60% of cases there was at least 50% obstruction and obstruction exceeded 80% in 25% of cases. It was also found that there was a positive correlation between CEAP classification and degree of obstruction (r = 0.330; p = 0.001), that the point of maximum compression was the proximal left common iliac vein in 70% of limbs, and that, in 30% of cases, other segments of the iliocaval venous system were compressed by adjacent arteries. In nine patients (18%), >50% IVOs were found bilaterally. The method achieved 94% sensitivity, 79.2% specificity, a 94% positive predictive value, a 79.1% negative predictive value, and 86.7% accuracy, while interobserver agreement was 92.1% (confidence interval [IC]: 87.1-97.7; kappa: 0.899).^29^^,^^30^ In addition to its high degree of accuracy for diagnosis of obstructions, CT can also be of help in identifying the point of greatest compression, the stent diameters and lengths needed, and even the best access route for treatment. Recently, we published our proposal for a classification of IVOs, which considers the point of greatest compression in the region of the confluence of the iliac veins and a caudal venous segment free from obstruction^30^ (Figures 2 and 3).

Very few studies have been published showing the capacity of magnetic resonance angiography for diagnosis of these obstructions and just one study has compared the method with IVUS.^31^ Massenburg et al. retrospectively compared magnetic resonance imaging with IVUS in 46 patients with clinical symptoms suggestive of IVO. They observed sensitivity of 100%, specificity of 22.7%, a positive predictive value of 58.5%, and a negative predictive value of 100%.^31^

Studies have demonstrated the superiority of IVUS in relation to venography for diagnosis of IVO.^22^^,^^24^^,^^29^^,^^32^ In addition to its capacity to determine the degree of mechanical compression, this is the only method capable of precisely detecting the presence of intraluminal obstructions (adhesions, trabeculae, and membranes), the characteristics of the wall, and the presence of residual thrombi and, most importantly, is able to precisely define the location and degree of reduction of the cross-sectional area of the vessel. In contrast, venography underestimates the degree of obstruction in approximately 30% of cases and can even fail to identify obstructions exceeding 50% in up to 25% of cases.^15^^,^^32^^,^^33^

**Figure 4:**
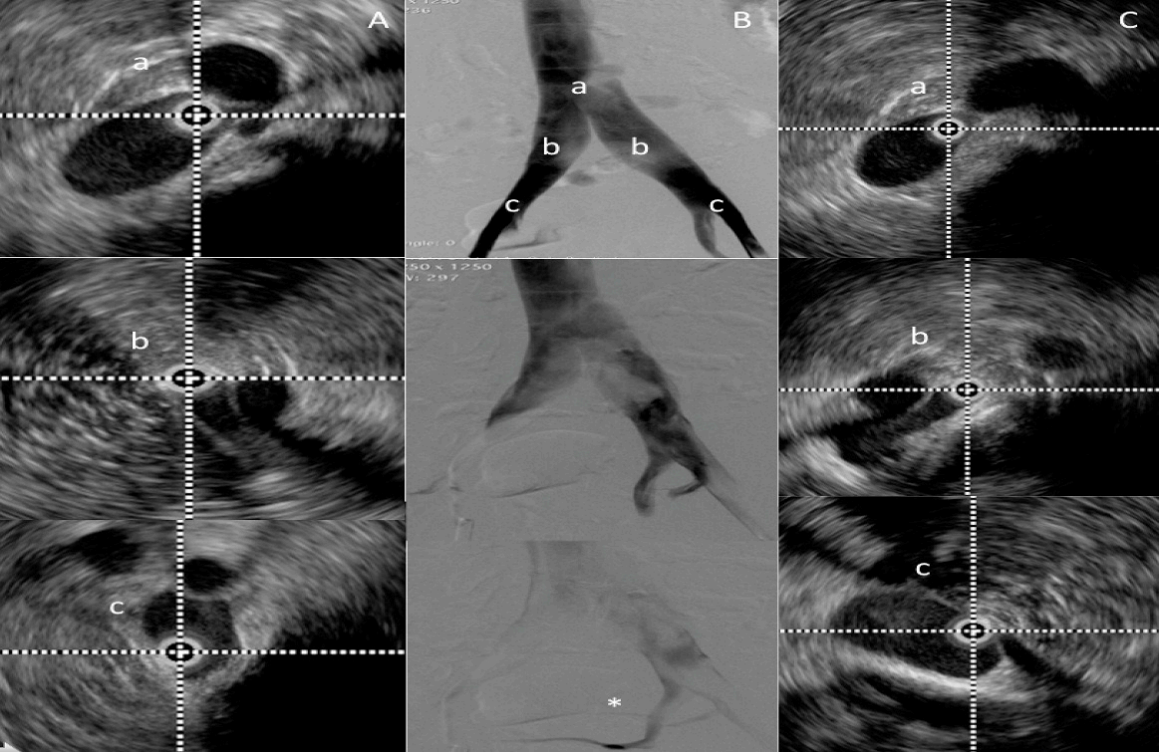
Venography and intravascular ultrasound for determination of degree of obstruction and segment obstructed: (A) Right limb; (B) Venography; (C) Left limb; *Confluence of the internal and external iliac veins; b- Common iliac vein; c- Confluence of the external and internal iliac veins.

Nowadays, IVUS is considered the gold standard for diagnosis of IVO. Some studies indicate sensitivity of 90% for diagnosis of these obstructions, especially in patients with advanced CVI. It is of fundamental importance for confirming and documenting the degree of obstruction, to determine the segment to be covered with the stent (adequate inflow and outflow) and, most of all, to detect residual obstruction and determine the success of treatment^29^^,^^32^^,^^33^ (Figure 4).

## TREATMENT TECHNIQUE

For endovascular treatment, use of digital subtraction, collimation, and the road-map technique is important to reduce the time exposed to radiation for the patient and the professionals in the operating room. It should be remembered that many of these patients are in the fertile age range and all precautions for protection against radiation should be taken.^34^ In the majority of cases, the procedure is conducted with local anesthesia and sedation only, but in cases employing popliteal and jugular accesses, in which the procedure can be uncomfortable, and in PTIVO, in which treatment of the occluded segment can be slow and painful, deep sedation or general anesthesia may be needed. It is recommended that the hormonal profile be assayed and serological pregnancy test or spermogram be requested for patients of fertile age.

Our preference is to obtain a retrograde access via the femoral vein, in the mid-third of the thigh ipsilateral to the obstruction, under ultrasound guidance. This access offers shorter and more direct access to the site of obstruction, enabling use of shorter catheters and guides, which in turn provides greater torque and maneuverability. Alternative access options in cases of femoral vein occlusion are the deep femoral vein, internal jugular vein, and popliteal vein. In the majority of cases, access via the groin is not appropriate, because it will often be necessary to extend the stent below the inguinal ligament. While the pressure inside the arteries limits use of sheaths larger than 6 or 7Fr, there is no such restriction with regard to the venous system. We most often use an 11Fr sheath.

Venography is the first imaging method used. We initially confirm adequate inflow, to determine the point at which the lower extremity of the stent should be positioned. It should not entrap the orifice of the deep femoral vein, but, in some cases, it may even be necessary to encroach upon this vein to guarantee adequate inflow.^30^ Venography is useful for identification of the length of the obstructed segment. However, it is not always possible and so multiplanar projections should always be used, investigating the presence of indirect signs, such as presence of collaterals. The anterograde venography technique, with Valsalva maneuver or balloon inflation, is sometimes necessary. Venography is useful when positive, but when it is negative, IVUS is indispensable to confirm absence of compressions and check for presence of intraluminal lesions (adhesions, membranes, and residual thrombi).

In the majority of cases, obstructions can be traversed without difficulty, using catheters and hydrophilic guidewires. In long occlusions, crossing the lesion may be very laborious, demanding multiple projections, knowledge of the anatomy, and use of many different catheter tips and profiles. In the venous system, it is not uncommon to encounter anatomic abnormalities. We always perform venous access under ultrasound guidance and bilateral venography. In all situations, but especially for long occlusions, when there is a rich network of collaterals, it is essential to inject contrast into the inferior vena cava (IVC) after crossing the obstructed segment, in order to confirm the position of the catheter inside this vessel. For reasons of safety, we always park the guidewire, with the aid of the catheter in the subclavian vein.

After venography, the IVUS catheter is inserted over the guidewire through the sheath. Using standard software, the diameters and area of the venous lumen should be measured in each segment (IVC, common iliac vein [CIV], external iliac vein [EIV], and femoral vein). The importance of IVUS can be highlighted by its capacity to determine the presence of diffuse intraluminal obstructions, membranes, and adhesions, which may extend beyond the point of maximum compression, particularly at the confluence of the iliac veins.

In our patients, we have seen that in 20 to 30% of postthrombotic syndrome (PTS) cases there is a combination of both focal and diffuse obstructions. In the arterial system, the degree of obstruction is calculated by comparing the focal lesion with the adjacent normal arterial lumen. This is acceptable because diffuse lesions are rare in the arteries. However, in the veins, diffuse lesions are so common (particularly in postthrombotic obstructions) that use of this method can lead to underestimation of the degree of obstruction. In the venous system, the degree of obstruction should be calculated using the value of the anatomic area considered normal for the segment in question (CIV: 200 mm^2^; EIV: 150 mm^2^; common femoral vein: 125 mm^2^).^35^ These values are required for drainage and, consequently, normal peripheral venous pressure.

It should be remembered that, while the accuracy of IVUS is superior to that of venography, it can still fail, particularly at the confluence of the iliac veins. At this point, the IVUS catheter may not be in a position coaxial with the axis of the vessel, causing the lumen in the opposite quadrant to appear elongated and dark, beyond the probe’s focal distance. If there are doubts with regard to analysis of the images, partial inflation of the angioplasty balloon, at low pressure, can be used to determine whether there are points of narrowing, or the preoperative CT images can be consulted to determine the exact point at which greatest compression occurs.^30^

As the IVUS catheter is advanced, presence, degree, and length of the obstruction should be observed and diameters and areas should be noted. While performing these measurements, the operator should determine the location at which the inferior extremity of the stent should be positioned, choosing a bony anatomic reference landmark, observed on fluoroscopy, below the most caudal obstruction. The operator should also mark the exact location of the confluence of the iliac veins, seen on IVUS, the point of maximum compression, and presence of any obstructive intraluminal structures beyond that point. Fluoroscopy should therefore be used to determine where the superior and inferior extremities of the stent should be positioned. The proximal edge of the stent may be placed at the superior, mid, or inferior part of the lumbar vertebrae, from the inferior margin of the L3 to the inferior margin of L5. In postthrombotic cases, it could be as high as the inferior margin of L2.

The IVUS catheter is removed and a balloon angioplasty catheter is used to dilate the entire obstructed venous segment. During this pre-dilation stage, we generally use conventional balloons, type XXL® (4 -5 ATM) (Boston Scientific, Natick, MA, United States), 16 mm, or 18 mm for PTS cases. There may be greater resistance to dilation in postthrombotic obstructions, occlusions, and lesions at points of compression and venous confluences. In these situations, it may be necessary to use low complacency, high pressure balloons, such as Atlas Gold® (6-18 ATM) (Bard, Tempe, United States). In some situations, pre-dilation with smaller-profile, low complacency, high pressure balloons may also be necessary, to enable progression of larger balloon catheters.

The balloon catheter is then removed and the stents are inserted to cover the entire area that has been dilated, preventing elastic recoil. Several stents may be needed to achieve this objective. We still prefer braided stents made from a cobalt-chromium-nickel-molybdenum alloy known as Elgiloy® (Wallstent®, Boston Scientific, Natick, United States). The majority of studies that have demonstrated long-term clinical efficacy and safety used this type of stent.^14^^,^^23^^,^^35^^,^^36^ Recent studies have confirmed the clinical applicability of dedicated venous nitinol stents, reporting very promising initial results, but we do not yet have conclusive follow-up studies of long-term patency.^37^^-^^40^

It is recommended that the entire obstructed segment should be covered, considering the measurements acquired with the angiotomography and/or IVUS conducted previously. Occlusion rates are not related to length or number of stents inserted, but to incomplete coverage of the obstructed segment, which can cause inadequate inflow or outflow. In general, stents with a diameter 2 mm larger than the diameter used for the pre-dilation balloon are preferred. The most commonly used stent diameters are 16, 18, and 20 mm. Woven braid stents shorten when post-dilated with the balloon catheter and so care must be taken to ensure that they will come to rest at the chosen anchor site. When it is necessary to use more than one stent, it is important to leave at least 3 to 5 cm overlap, for the same reason. Occurrence of angles will increase the probability of re-obstruction in areas with tight bends; at this point it may be necessary to overlap even further.

There is currently controversy with relation to the extent to which the stent should protrude into the IVC.^41^^,^^42^ According to the original technique as described by Raju and Neglen^12^^,^^15^^,^^21^^,^^23^, and still used today by the great majority of authors, the Wallstent® is positioned at least 2 to 3 cm into this vessel. According to the same authors, when this precaution is not observed, proximal re-obstruction occurs in 40% of cases, because of crushing and distal migration of the stent.^15^^,^^43^^,^^44^ This technique is extremely important, because in more than half of cases the point of maximum compression is located at or above the confluence.^45^^-^^47^ In our patients, we found that the obstruction occurred below the confluence of the iliac veins in 41.6% of cases, at the level of confluence in 34.5%, and above it, within the IVC, in 23.9%.^30^

When using the technique of inserting the Wallstent® into the vena cava, it is possible to trap the contralateral iliac vein, causing venous thrombosis. Clinical studies and metanalyses have reported contralateral DVT in 1.1 to 2.2% of cases.^14^^,^^23^ In a randomized study^4^ and in our accumulated clinical experience of more than 250 cases treated, we observed just two cases of DVT in the contralateral limb (1%), both in patients with a clinical history of prior DVT and who did not follow the recommended oral anticoagulant regimen.

Recently, Murphy et al.,^41^ conducted a retrospective study comparing the technique using a Wallstent® in isolation against insertion of the Cook-Z-Stent® (Cook Medical, Bloomington, United States) combined with a Wallstent® (Boston Scientific, Natick, United States), which is a hybrid technique proposed by Raju et al.^48^ They observed cumulative contralateral patency of 99 and 90% in the Z-Stent® and Wallstent® groups, respectively (p<0.001), after 5 years of clinical follow-up. However, the study is subject to some important limitations. The group treated with Z-Stents® was compared with a historical series of cases; therefore the experience acquired may have influenced patient selection, techniques, and results, making the two groups imperfectly comparable.

Caliste et al.^49^ conducted a retrospective study that investigated the incidence of contralateral iliac DVT after using stents that cross the iliocaval confluence. They studied 41 cases, 39 (95%) of which had postthrombotic obstructions, and in 22 (54%) the obstructions involved the IVC. Four patients (9.7%) developed contralateral DVT, three of whom had inadequate anticoagulation. Just 2.4% of the patients who were adequately anticoagulated developed contralateral DVT (p = 0.0004).

**Figure 5:**
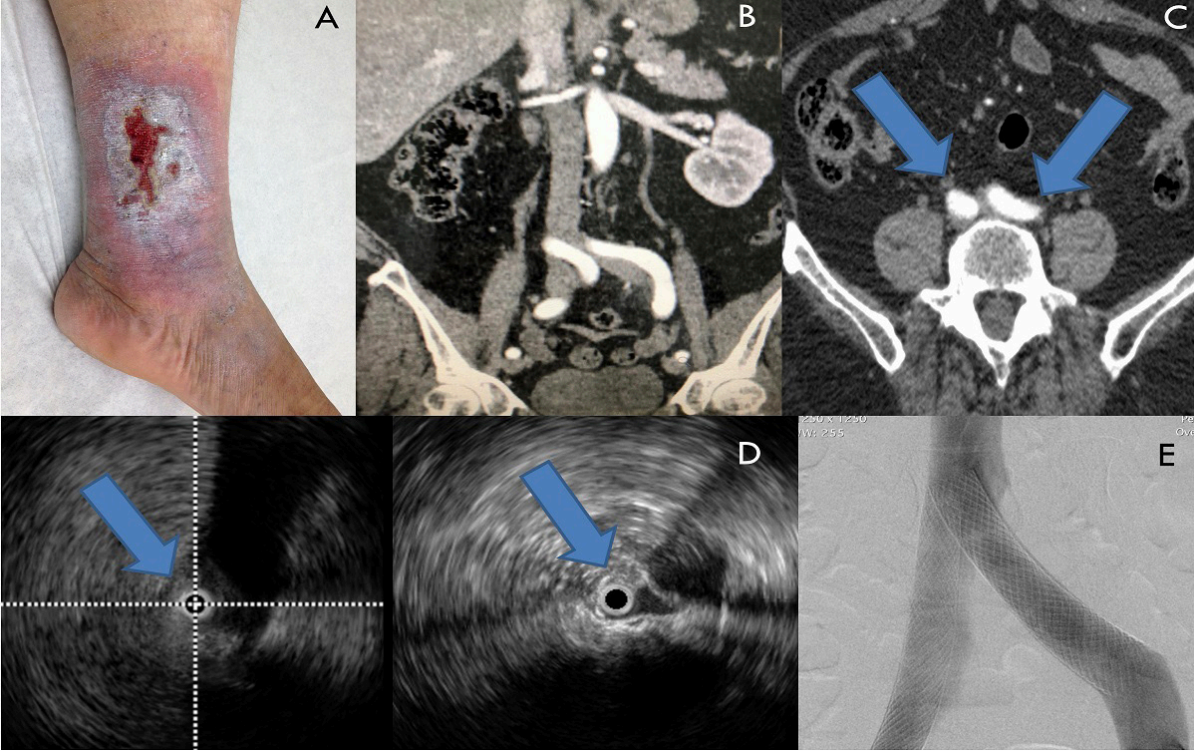
Angioplasty with bilateral stenting in a patient with clinical, etiological, anatomic, and pathophysiological classification (CEAP) classification grade C6, with a severe obstruction compromising the iliac veins bilaterally: (A) Unilateral venous ulcer (chronic venous insufficiency, CEAP C6S); Presence of severe bilateral iliac compression: (B, C) Angiotomography; (D) intravascular ultrasound, confirming severe bilateral obstruction (>50%); (E) Satisfactory result with stent deployment using the double-barrel technique.

It should be remembered that bilateral compression or obstruction is not uncommon, particularly not in patients with PTS. It is known that venography and, sometimes, even IVUS can fail to show concomitant obstructions, especially at points of confluence, which can limit adequate inflow and increase the likelihood of venous thrombosis. This is why it is recommended that a multiplanar study of the angiotomographic images is performed preoperatively, correcting the angles of the vessels studied, in order to enable detection of points of compression of the iliocaval axis bilaterally; which should then be confirmed with IVUS intraoperatively. Z-Stents are not available at our institution and we therefore still use the original technique proposed by Raju et al.^12^^,^^15^^,^^21^^,^^23^. In cases in which there is bilateral obstruction exceeding 50% (confirmed by IVUS) and/or presence of compression of the IVC, which occurred in 11% of the cases in our series, we chose to use the parallel stents technique (double barrel), a technique initially proposed by Neglén et al.^47^ (Figure 5).

**Figure 6:**
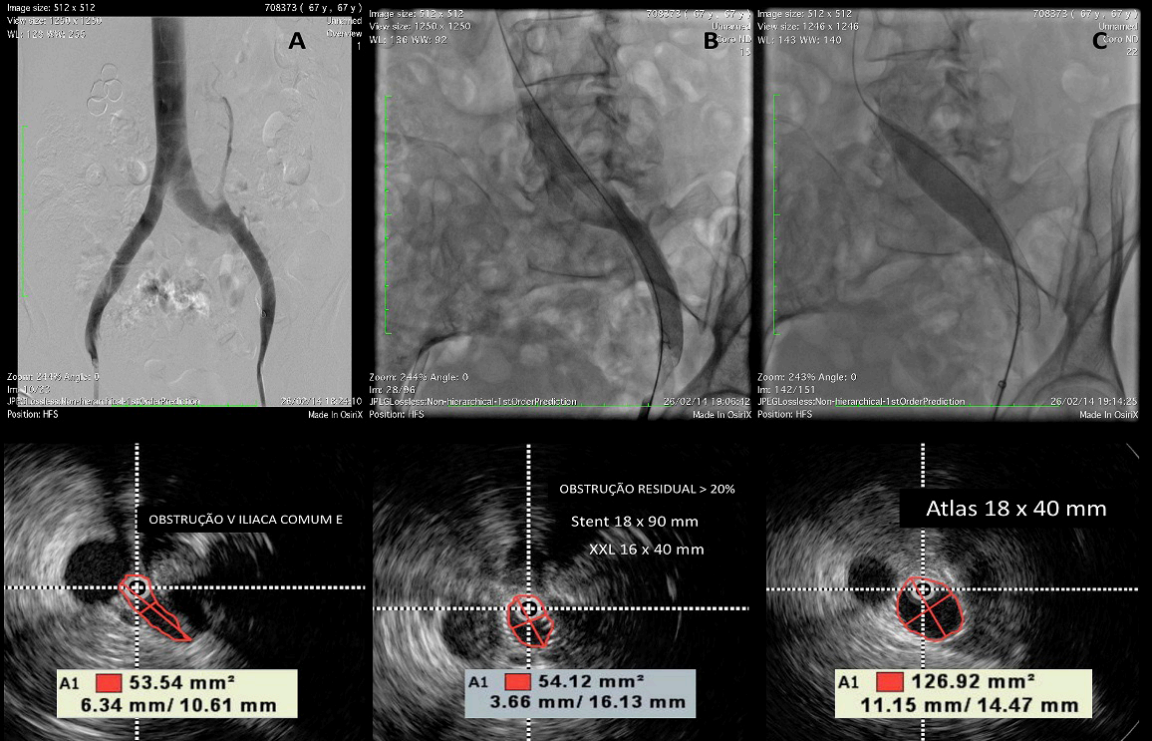
Final control intravascular ultrasound (IVUS). In (A), initial phlebography demonstrating direct and indirect signs of obstruction of the left iliac vein, confirmed by IVUS (area at the point of greatest compression is 53.5 mm^2^). In (B), control phlebography apparently demonstrating a satisfactory result, but not confirmed by IVUS (area at the point of greatest compression is 54.1 mm^2^). In (C), final result after angioplasty with low complacency, high pressure balloon.

The balloon catheter is then reintroduced for post-dilation of the stent. We used the same 16 mm balloons as routine, and 18 mm balloons for PTS cases, taking great care when advancing and withdrawing them, which should always be performed with the balloon completely deflated, inserting it with a rotating movement, without resistance, to avoid perforation of the mesh and stent displacement. Checks should always be made for lesions upstream or downstream of the edges of the stent and, most importantly, the final area of the venous lumen should be checked, to ensure that it meets the minimum anatomic parameters described previously, and IVUS is the only method that can be used to do this precisely.^50^^,^^51^ The lumen should be regularly shaped and the stent should be well affixed to the vein wall. Sometimes, in cases of PTS that are difficult to dilate, it may be necessary to use high-pressure balloons. It should be remembered that experimental studies indicate that residual obstructions of 20% can be enough to maintain the ectasia and the symptoms, resulting in clinical failure of the treatment.^51^ In our patients, in 30% of cases venography failed to diagnose residual obstruction when compared to IVUS, highlighting the importance of IVUS for successful treatment (Figure 6).

Venography is the method to use for final evaluation of results. The lumen of the segment treated should be free from irregularities or obstructions. Collaterals should disappear, because the principal channel for drainage of flow is of lower resistance, although when they have been present for a long time and are very dilated, it is not uncommon for them to still be partially visible on the final venography, but flow should be greater and preferential via the main channel, and contralateral vessels should not contain blocked flow. After removal of the introducer sheath, manual compression is applied to the puncture site.

## USE OF ANTITHROMBOTICS

During the preoperative period for MTS or NTIVO interventions, we administer acetylsalicylic acid (ASA) 100 mg/d and clopidogrel 75 mg/d, starting 7 days before the procedure. Patients with PTIVO or CS who are no longer taking oral anticoagulants are managed in the same manner. Those who are taking anticoagulants are instructed to suspend them for the necessary period, so that their effects will subside. In the case of warfarin, an international normalized ratio (INR) of 1.5-2.0 is targeted on the day of angioplasty. Intraoperatively, patients are given a minimum of 5,000 U of unfractionated heparin (100 U/kg). While still in hospital, they are kept on full anticoagulation with low molecular weight heparin.

After hospital discharge, which in the majority of cases takes place on the day or morning after intervention in cases of MTS/NTIVO (when the procedure is judged a technical success), clopidogrel 75 mg/d combined with ASA 100 mg/d is prescribed for 6 months and then ASA 100 mg/d for the rest of the patient’s life. For patients with CS/PTIVO and recanalization of long occlusions, anticoagulation is recommended with anti-vitamin K and strict control of the INR, or with new direct oral anticoagulants, for a minimum period of 6 months. Patients who were already taking these medications preoperatively (for thrombophilias and recurrent thromboses) will be kept anticoagulated indefinitely during the postoperative period. In our experience, this type of protocol has been associated with good response to treatment and long-term patency, but it must be remembered that there are not yet any studies that have tested which antithrombotic treatment regime is the best in this subset of patients.^4^

## POSTOPERATIVE FOLLOW-UP

As has been mentioned above, at the initial consultation we evaluate and record the intensity of pain using a VAPS, assess CEAP and VCSS clinical classifications, administer the SF-36 quality of life questionnaire to our patients, and record the diameters of limbs at the mid thigh, leg, and mid-foot. These data are collected again at postoperative consultations, which are routinely scheduled for 1, 3, 6, and 12 months and annually thereafter. At these visits, the patient is also examined with venous color Doppler ultrasonography and the same parameters described above are evaluated.^7^ At the 6-month and 1-year visits we also request X-rays of the pelvis to confirm the integrity and position of the stent. In asymptomatic patients, if there is a suspicion of intra-stent obstruction on Doppler ultrasonography, or if there is a relapse of symptoms, the patient undergoes angiotomography and venography with insertion of the IVUS catheter once more, with the option to dilate the point of obstruction with a balloon catheter that has the same diameter as the stent. In some cases, it may be necessary to deploy another stent proximal or distal of the treated segment.

## RESULTS

Several different retrospective studies, meta-analyses, and medical society guidelines suggest that placement of an iliac stent is effective and safe and should be considered the treatment of choice for patients with iliac obstructions, associated or not with reflux, and for patients with severe symptoms and high CEAP classification grades.^14^^,^^23^^,^^36^^,^^52^^,^^53^

In a recent, double-blind, randomized study comparing clinical and endovascular treatment in patients with iliac vein obstruction and high grade CEAP classifications, we found that 28% of patients in follow-up at a venous insufficiency clinic met inclusion criteria for the study and that 60% of the members studied had ≥50% iliac obstruction on IVUS. This shows the high prevalence of this clinical status at a specialist tertiary hospital, considering that on this protocol only patients with CEAP C3-6 who were in follow-up for more than 1 year without response to treatment were investigated. The immediate technical success rate was 100% and there were no serious perioperative complications. After 6 months’ follow-up, the median VAPS scores reduced from 8 to 2.5 in patients fitted with stents, and from 8 to 7 in patients who only received clinical treatment (p < 0.001). The VCSS reduced from a median of 18.5 to 11 in the group treated with stents, and from 15 to 14 in the group on clinical treatment (p < 0.001). Median total scores on the SF-36 quality of life questionnaire increased from 53.9 to 85.0 with stent placement and from 48.3 to 59.8 after clinical treatment (p < 0.001). There were no stent fractures or migration and primary, primary assisted, and secondary patency rates were 92%, 96%, and 100%, respectively (median: 11.8 months; range: 6-18 months).^4^

Few studies have investigated clinical outcomes exclusively in patients with MTS or NTIVO. The majority of studies mix thrombotic and non-thrombotic obstructions and many include treatment of superficial venous reflux. Apparently, this group of patients has favorable clinical outcomes, particularly in terms of relief from pain and cure of venous ulcers.

Raju and Neglén^54^ observed that 2.5 years after fitting stents there was complete relief from pain, complete resolution of swelling, and sustained healing of ulcers in 77%, 53%, and 76%, respectively. In a later study, they found a 62% rate of sustained ulcer healing after 5 years of clinical follow-up.^53^ Ye et al. reported rates of pain relief, resolution of swelling, and ulcer healing of 87%, 88%, and 74%, respectively, in 101 limbs with mean follow-up of 4 years.^55^ Quality of life scores improved significantly in both studies. Raju and Neglén^54^ described a similar result in subsets of limbs with NTIVO alone and associated with reflux, even when this was left untreated.

Another interesting study was conducted by Meng et al.^56^ who showed that, in the presence of iliac obstruction, just 13% of limbs with operated varicose veins had significant relief from symptoms after 2 months’ follow-up. While cumulative patency is lower among patients with PTIVO, the results are nevertheless highly satisfactory. Neglen et al. observed primary, primary assisted, and secondary patency of 57%, 80%, and 86%, respectively, after 5 years’ follow-up.^53^ Studies show that secondary patency at mean follow-up of 4 to 7 years is in the range of 74% to 89%.^23^ Patients treated for chronic occlusion have recanalization rates of 83% to 95%.^14^^,^^23^ Raju and Néglen^57^ observed 66% secondary patency in 139 limbs treated for chronic occlusion at 4-year follow-up and rates of relief from pain and resolution of edema of 79% and 66%, respectively. In a study that reported recanalizations of total and long obstructions in the femoral-iliocaval segment, secondary patency was 66% to 89% with follow-up of 4 to 7 years.^23^ Ulcer healing rates are lower in postthrombotic patients than in the non-thrombotic cohort, but even in this subset, a 60% cumulative rate of ulcer healing was seen at 5 years.^58^ In patients with ulcers that do not heal, the possibility of intra-stent obstruction and reflux in the superficial and perforating vein system should be investigated.

## CONCLUSIONS

Iliac vein obstruction is highly prevalent, particularly among patients with advanced CVI. Although it can be asymptomatic, it is frequently associated with incapacitating symptoms and considerable impairment of quality of life. Its presence remains little investigated and the lack of well-established criteria for noninvasive diagnosis is a contributing factor in failure to properly diagnose and adequately treat many patients. Endovascular treatment is currently considered the gold standard. It can be performed with high technical success rates, low morbidity and mortality, and high rates of patency and therapeutic success, as long as the anatomic and pathophysiologic characteristics that occur in the presence of these obstructions are taken into consideration.

## References

[1] Cushman M (2007). Epidemiology and risk factors for venous thrombosis. Semin Hematol.

[2] Raju S (2013). Best management options for chronic iliac vein stenosis and occlusion. J Vasc Surg.

[3] Rossi FH, Kambara AM, Izukawa NM (2018). Randomized double-blinded study comparing medical treatment versus iliac vein stenting in chronic venous disease. J Vasc Surg Venous Lymphat Disord.

[4] Rossi FH, Kambara AM, Izukawa NM (2015). Randomized double-blinded study comparing clinical versus endovascular treatment of iliac vein obstruction: preliminary results. J Vasc Surg.

[5] Rossi FH, Kambara A, Pinto I (2016). Efficacy of Computed Tomography Venography (CTV) Screening Compared to Duplex Ultrasound (DU), Multiplanar Venography (MV), and Intravascular Ultrasound (IVUS) in Iliac Vein Compression Syndrome (IVCS). J Vasc Surg.

[6] Rossi FH, Gama CAR, Fonseca IYI (2014). Computed Tomograpy Venography diagnosis of iliocaval venous obstruction in advanced chronic venous insufficiency. J Vasc Bras.

[7] Metzger PB, Rossi FH, Kambara AM (2016). Criteria for detecting significant chronic iliac venous obstructions with duplex ultrasound. J Vasc Surg.

[8] Abboud G, Midulla M, Lions C (2010). “Right-sided” May-Thurner syndrome. Cardiovasc Intervent Radiol.

[9] Cockett FB, Thomas ML, Negus D (1967). Iliac vein compression: Its relation to iliofemoral thrombosis and the post-thrombotic syndrome. BMJ.

[10] Wu M-K, Luo X-Y, Zhang F-X (2016). Incidence and risk factors of deep venous thrombosis in asymptomatic iliac vein compression: a prospective cohort study. Chin Med J (Engl).

[11] Mavor GE, Galloway JM (1967). Collaterals of the deep venous circulation of the lower limb. Surg Gynecol Obstet.

[12] Neglén P, Thrasher TL, Raju S (2003). Venous outflow obstruction: an underestimated contributor to chronic venous disease. J Vasc Surg.

[13] Garg N, Gloviczki P, Karimi KM (2011). Factors affecting outcome of open and hybrid reconstructions for nonmalignant obstruction of iliofemoral veins and inferior vena cava. J Vasc Surg.

[14] Seager MJ, Busuttil A, Dharmarajah B, Davies AH (2016). A systematic review of endovenous stenting in chronic venous disease secondary to iliac vein obstruction. Eur J Vasc Endovasc Surg.

[15] Neglén P, Berry MA, Raju S (2000). Endovascular surgery in the treatment of chronic primary and post-thrombotic iliac vein obstruction. Eur J Vasc Endovasc Surg.

[16] Daugherty SF, Gillespie DL (2015). Venous angioplasty and stenting improve pelvic congestion syndrome caused by venous outflow obstruction. J Vasc Surg.

[17] Huskisson EC, Jones J, Scott PJ (1976). Application of visual-analogue scales to the measurement of functional capacity. Rheumatol Rehabil.

[18] Eklöf B, Rutherford RB, Bergan JJ (2004). Revision of the CEAP classification for chronic venous disorders: consensus statement. J Vasc Surg.

[19] Passman MA, McLafferty RB, Lentz MF (2011). Validation of Venous Clinical Severity Score (VCSS) with other venous severity assessment tools from the American Venous Forum, National Venous Screening Program. J Vasc Surg.

[20] Lozano Sánchez FS, Sánchez Nevarez I, González-Porras JR (2013). Quality of life in patients with chronic venous disease: influence of the socio-demographical and clinical factors. Int Angiol.

[21] Neglén P, Raju S (1993). Detection of outflow obstruction in chronic venous insufficiency. J Vasc Surg.

[22] Almeida BL, Rossi FH, Sousa AGMR (2018). Correlation between venous pressure gradients and intravascular ultrasound in the diagnosis of iliac vein compression syndrome. J Vasc Surg.

[23] Raju S (2013). Best management options for chronic iliac vein stenosis and occlusion. J Vasc Surg.

[24] Gagne PJ, Gasparis A, Black S (2018). Analysis of threshold stenosis by multiplanar venogram; intravascular ultrasound examination for predicting clinical improvement after iliofemoral vein stenting in VIDIO trial. J Vasc Surg.

[25] Labropoulos N, Borge M, Pierce K, Pappas PJ (2007). Criteria for defining significant central vein stenosis with duplex ultrasound. J Vasc Surg.

[26] Bauer AR, Flynn RR (1988). Computed tomography diagnosis of venous thrombosis of the lower extremities and pelvis with contrast material. Surg Gynecol Obstet.

[27] Chung JW, Yoon CJ, Jung SI (2004). Acute Iliofemoral Deep Vein Thrombosis: Evaluation of Underlying Anatomic Abnormalities by Spiral CT Venography. J Vasc Interv Radiol.

[28] Oguzkurt L, Tercan F, Pourbagher MA, Kizilkilic O, Turkoz R, Boyvat F (2005). Computed tomography findings in 10 cases of iliac vein compression (May–Thurner) syndrome. Eur J Radiol.

[29] Rossi FH, Kambara A, Pinto I (2016). Efficacy of Computed Tomography Venography (CTV) Screening Compared to Duplex Ultrasound (DU), Multiplanar Venography (MV), and Intravascular Ultrasound (IVUS) in Iliac Vein Compression Syndrome (IVCS). J Vasc Surg.

[30] Rossi FH, Kambara AM, Rodrigues TO, Rossi CBO, Izukawa NM, Pinto IMF, Thorpe PE (2020). Comparison of computed tomography venography and intravascular ultrasound in screening and classification of iliac vein obstruction in patients with chronic venous disease. J Vacs Surg: Venous and Lym Dis.

[31] Massenburg BB, Himel HN, Blue RC, Marin ML, Faries PL, Ting W (2015). Magnetic resonance imaging in proximal venous outflow obstruction. Ann Vasc Surg.

[32] Neglén P, Raju S (2002). Intravascular ultrasound scan evaluation of the obstructed vein. J Vasc Surg.

[33] Raju S (2008). Endovenous treatment of patients with iliac-caval venous obstruction. J Cardiovasc Surg (Torino).

[34] Fidalgo Domingos L, San Norberto García EM, Gutiérrez Castillo D, Flota Ruiz C, Estévez Fernández I, Vaquero Puerta C (2018). Radioprotection measures during the learning curve with hybrid operating rooms. Ann Vasc Surg.

[35] Murphy E, Nguyen D, Varney E, Stears C, Raju S (2015). Increasing the diagnostic sensitivity of noninvasive imaging techniques before and after iliac vein stenting. J Vasc Surg.

[36] Razavi MK, Jaff MR, Miller LE (2015). Safety and effectiveness of stent placement for iliofemoral venous outflow obstruction: systematic review and meta-analysis. Circ Cardiovasc Interv.

[37] Razavi M, Marston W, Black S, Bentley D, Neglen P (2018). The initial report on 1-year outcomes of the feasibility study of the veniti vici venous stent in symptomatic iliofemoral venous obstruction. J Vasc Surg.

[38] de Wolf MAF, de Graaf R, Kurstjens RLM, Penninx S, Jalaie H, Wittens CHA (2015). Short-term clinical experience with a dedicated venous nitinol stent: initial results with the sinus-venous stent. Eur J Vasc Endovasc Surg.

[39] Stuck AK, Kunz S, Baumgartner I, Kucher N (2017). Patency and clinical outcomes of a dedicated, self-expanding, hybrid oblique stent used in the treatment of common iliac vein compression. J Endovasc Ther.

[40] Lichtenberg M, Stahlhoff WF, Özkapi A, de Graaf R, Breuckmann F (2019). Safety, procedural success and outcome of the Aspirex®S endovascular thrombectomy system in the treatment of iliofemoral deep vein thrombosis - data from the Arnsberg Aspirex registry. Vasa.

[41] Murphy E, Johns B, Alias M, Crim W, Raju S (2017). Deep venous thrombosis associated with caval extension of iliac stent. J Vasc Surg Venous Lymphat Disord.

[42] Gloviczki P, Lawrence PF (2017). Iliac vein stenting and contralateral deep vein thrombosis. J Vasc Surg.

[43] Raju S, Owen S, Neglén P (2002). The clinical impact of iliac venous stents in the management of chronic venous insufficiency. J Vasc Surg.

[44] Raju S, McAllister S, Neglén P (2002). Recanalization of totally occluded iliac and adjacent venous segments. J Vasc Surg.

[45] Ehrich WE, Krumbhaar EB (1943). A frequent obstructive anomaly of the mouth of the left common iliac vein. Am Heart J.

[46] Ang WC, Doyle T, Stringer MD (2013). Left-sided and duplicate inferior vena cava: a case series and review. Clin Anat.

[47] Neglén P, Darcey R, Olivier J, Raju S (2010). Bilateral stenting at the iliocaval confluence. J Vasc Surg.

[48] Raju S, Ward M, Kirk O (2014). A modification of iliac vein stent technique. Ann Vasc Surg.

[49] Caliste XA, Clark AL, Doyle AJ, Cullen JP, Gillespie DL (2013). The incidence of contralateral iliac venous thrombosis after stenting across the iliocaval confluence in patients with acute or chronic venous outflow obstruction. J Vasc Surg Venous Lymphat Disord.

[50] Raju S, Buck WJ, Crim W, Jayaraj A (2018). Optimal sizing of iliac vein stents. Phlebology.

[51] Raju S, Kirk O, Davis M, Olivier J (2014). Hemodynamics of “critical” venous stenosis and stent treatment. J Vasc Surg.

[52] Wen-da W, Yu Z, Yue-xin C (2016). Stenting for chronic obstructive venous disease: a current comprehensive meta-analysis and systematic review. Phlebology.

[53] Neglén P, Hollis KC, Olivier J, Raju S (2007). Stenting of the venous outflow in chronic venous disease: long-term stent-related outcome, clinical, and hemodynamic result. J Vasc Surg.

[54] Raju S, Neglén P (2006). High prevalence of nonthrombotic iliac vein lesions in chronic venous disease: a permissive role in pathogenicity. J Vasc Surg.

[55] Ye K, Lu X, Li W (2012). Long-term outcomes of stent placement for symptomatic nonthrombotic iliac vein compression lesions in chronic venous disease. J Vasc Interv Radiol.

[56] Meng Q-Y, Li X-Q, Qian A-M, Sang H-F, Rong J-J, Zhu L-W (2011). Endovascular treatment of iliac vein compression syndrome. Chin Med J (Engl).

[57] Raju S, Neglén P (2009). Percutaneous recanalization of total occlusions of the iliac vein. J Vasc Surg.

[58] Raju S, Kirk OK, Jones TL (2013). Endovenous management of venous leg ulcers. J Vasc Surg.

