# Supplementary material for: Best practices in diagnosis and treatment of chronic iliac vein obstruction
Source: J Vasc Bras. 2020 Sep 14;19:e20190134. doi: 10.1590/1677-5449.190134 (PMC8276648; doi:10.1590/1677-5449.190134)
Supplement: Translation

# Translation: O caminho das pedras no diagnóstico e tratamento da síndrome obstrutiva ilíaca venosa crônica

**Authors:** Fabio Henrique Rossi, Thiago Osawa Rodrigues, Nilo Mitsuru Izukawa, Antônio Massamitsu Kambara

**Affiliations:** 1 Instituto Dante Pazzanese de Cardiologia de São Paulo – IDPC-SP, São Paulo, SP, Brasil.; 2 Centro de Intervenções Endovasculares – CIEV-IDPC, São Paulo, SP, Brasil.

**Keywords:** síndrome de May-Thurner, síndrome de Cockett, veia ilíaca, trombose venosa, angioplastia, estenose, stent

## Abstract

A obstrução venosa ilíaca ocorre em 20% a 30% da população. Nos portadores de insuficiência venosa crônica grave, essa prevalência é ainda maior, podendo chegar a 50% a 90% dos pacientes, situação em que essa obstrução é investigada pelo ultrassom intravascular. Métodos diagnósticos menos invasivos, como o Doppler vascular, ou mesmo invasivos, como a flebografia, podem falhar em seu diagnóstico. O tratamento endovascular dessas obstruções tem se demonstrado eficaz, seguro e associado a excelente resultado clínico e de perviedade, desde que princípios anatômicos e técnicos fundamentais sejam considerados e aplicados.

## INTRODUÇÃO

A obstrução venosa ilíaca (OVI) pode ser classificada em primária, obstrução não trombótica de veia ilíaca (ONTVI), classicamente chamada de síndrome de May-Thurner (SMT); ou secundária, obstrução pós-trombótica de veia ilíaca (OPTVI), ou síndrome de Cockett (SC)^1^. Seu tratamento endovascular, hoje considerado o padrão-ouro, está associado a melhora clínica e da qualidade de vida, a baixa morbimortalidade e a altos índices de perviedade^2^ ^-^ ^4^. Nos casos de insuficiência venosa com classificação clínica, etiológica, anatômica e patofisiológica (CEAP) entre C3 e C6, a ultrassonografia intravascular (UI) pode detectar obstrução da veia ilíaca em 50 a 90% dos membros investigados^2^ ^,^ ^4^ ^,^ ^5^. Apesar de a compressão ocorrer mais frequentemente no ponto de cruzamento entre a artéria ilíaca direita e a veia ilíaca esquerda, não é incomum que ela ocorra em outros segmentos. Em nossa casuística, e nas de outros autores, 30% das obstruções identificadas foram observadas em outros pontos que não aquele classicamente descrito^6^ ^-^ ^8^. Essa compressão pode provocar, além da redução da área de secção transversal do vaso, a formação de membranas e de traves fibroblásticas, e também trombose^9^ ^-^ ^11^ [60% das obstruções, em nossa casuística, apresentavam episódio prévio de trombose venosa profunda (TVP)]^4^. Na TVP associada à compressão do eixo cavo-ilíaco-femoral, frequentemente ocorre fleboesclerose (fenômeno de Rokitansky), retração cicatricial e obstruções extensas. Devemos lembrar que em apenas 20 a 30% dos casos verifica-se revascularização completa da luz do vaso acometido^11^. Outras causas menos comuns de compressão são: tumores benignos e malignos, fibrose retroperitoneal, lesões iatrogênicas, irradiação, cistos e aneurismas.

Todas as manifestações clínicas relacionadas à insuficiência venosa crônica (IVC) podem ser causadas por lesões obstrutivas e pela consequente hipertensão venosa. Dor crônica intratável, claudicação venosa e edema são importante sinais e sintomas preditivos da presença dessas obstruções^1^ ^,^ ^9^ ^,^ ^12^ ^,^ ^13^. Há uma associação positiva entre gravidade dos sintomas, classificação clínica, e grau da OVI (p = 0,001)^6^. A associação entre a obstrução e o refluxo venoso parece estar relacionada aos casos clínicos mais graves^13^ ^-^ ^15^. Tem sido observado que a OVI também pode estar relacionada à síndrome de congestão pélvica^16^, presente em 26% de nossa casuística.

## AVALIAÇÃO CLÍNICA

Na visita inicial, deve-se verificar a intensidade da dor, utilizando a escala visual analógica da dor (EVAD)^17^ e a classificação CEAP^18^; mensurar os diâmetros dos membros no meio da coxa, perna e mediopé; classificar o membro pelo *Venous Clinical Severity Score* (VCSS)^19^; e submeter os pacientes ao questionário de qualidade de vida SF-36^20^. É importante verificar a gravidade e, sobretudo, observar a evolução clínica e a resposta dos pacientes ao tratamento. Em nossa prática clínica, aos pacientes que apresentam IVC com CEAP ≥ C3, EVAD ≥ 3, VCSS ≥ 8, considerável comprometimento da qualidade de vida e obstrução ilíaca ≥ 50%, oferecemos o tratamento endovascular do território obstruído.

## FISIOPATOLOGIA

Uma série de diferenças anatômicas, fisiológicas e mecânicas existentes entre o sistema venoso e o arterial deve ser considerada para que seja obtido o sucesso no tratamento. O sistema venoso é convergente e apresenta baixa velocidade, alto volume de fluxo e baixa pressão, além de ser altamente complacente. Uma pequena elevação na pressão venosa capilar pode ser responsável pelo aparecimento de sinais e sintomas. A descompressão ou desobstrução do sistema venoso profundo e a consequente diminuição da ectasia e da hipertensão venosa é o princípio básico e fundamental do tratamento. Devemos lembrar que a veia ilíaca é a principal via de escoamento do fluxo venoso oriundo dos membros inferiores. Entretanto, ainda hoje, não se sabe a partir de que grau ou extensão a obstrução se torna hemodinamicamente significativa. Estudos que verificaram medidas diretas de pressão demonstraram-se inconclusivos^21^ ^,^ ^22^.

## MÉTODOS DIAGNÓSTICOS

A experiência clínica demonstra que o tratamento de obstruções ≥ 50% está relacionado a melhora dos sintomas e da qualidade de vida e, dessa forma, considera-se que essas obstruções são hemodinamicamente significativas e devem ser tratadas^4^ ^,^ ^14^ ^,^ ^23^. Como não existe exame hemodinâmico confiável, o diagnóstico e o tratamento vêm sendo realizados considerando a análise morfológica das obstruções. A venografia, que por muito tempo foi considerada o método padrão-ouro, pode ser uma boa ferramentadiagnóstica na presença de obstruções graves, mas falha quando comparada ao ultrassom intravascular (*intravascular ultrasound*, IVUS) em ao menos 1/3 dos casos^4^ ^,^ ^24^. Os sinais venográficos indiretos que sugerem a presença de OVI são: alargamento da veia ilíaca (*pancaking*), rarefação do contraste central (sinal do alvo ou *bull’s-eye*), e presença de colaterais transpélvicas ou paravertebrais (Figura 1).

**Figura 1:** Imagens flebográficas por subtração digital de pacientes portadores de obstruções ilíacas: (A) Alargamento (*Pancacking*) e compressão em segmento proximal de veia ilíaca comum esquerda; (B) Compressão severa em segmento proximal da veia ilíaca comum esquerda e presença de colateral paravertebral; (C) Presença de rarefação central de contraste em segmento proximal de veia ilíaca comum esquerda (sinal do alvo ou *bull’s-eye*); (D) Compressão bilateral de veias ilíacas comuns proximais; (E) Presença de membranas intraluminais em veia ilíaca comum esquerda; (F) Obstrução severa de veia ilíaca esquerda com formação de rede de colaterais paravertebral e transpélvica.

O eco-Doppler venoso colorido faz parte da investigação inicial dos pacientes portadores de IVC. É um método não invasivo e de baixo risco. Entretanto, é operador dependente, e mesmo em laboratórios especializados pode falhar em até 20% dos casos^7^ ^,^ ^25^. Em nossa instituição, a comparação de medidas diretas e indiretas obtidas por meio desse método com aquelas obtidas pelo IVUS mostrou que a razão de velocidade ≥ 2,5 através do ponto de maior obstrução foi o melhor parâmetro para o diagnóstico de obstrução ≥ 50% quando comparado ao IVUS (r = 0,790; p < 0,001). Nesse estudo foi proposto um algoritmo que atingiu a acurácia de 86,7% para o diagnóstico ultrassonográfico dessas obstruções (k = 0,73; p < 0,001)^7^.

A utilidade da tomografia computadorizada (TC) no diagnóstico da TVP foi amplamente descrita^26^ ^-^ ^29^; entretanto, pouquíssimos estudos verificaram sua acurácia na identificação das OVIs crônicas. Ao avaliarem o poder diagnóstico da TC em verificar a presença e o grau de obstruções ilíacas por meio de reformatações multiplanares 3D das imagens, quando comparado ao da IVUS em pacientes portadores de IVC avançada (CEAP C3-6), Rossi et al. observaram que em 60% dos casos havia ao menos 50% de obstrução, e que em 25% dos casos a obstrução era superior a 80%. Verificou-se também que havia uma correlação positiva entre a classificação CEAP e o grau de obstrução (r = 0,330; p = 0,001), que o ponto de máxima compressão foi a veia ilíaca esquerda comum proximal em 70% dos membros, e que, em 30% dos casos, outros segmentos do sistema de venoso cavo-ilíaco foram comprimidos pelas artérias adjacentes. Em nove pacientes (18%), foram encontradas OVIs >50% bilaterais. O método obteve sensibilidade de 94%, especificidade de 79,2%, valor preditivo positivo de 94%, valor preditivo negativo de 79,1%, acurácia de 86,7%, e apresentou uma concordância interobservador de 92,1% [intervalo de confiança (IC): 87,1-97,7; kappa: 0,899]^29^ ^,^ ^30^. A TC, além de possuir uma alta acurácia no diagnóstico das obstruções, pode auxiliar também na determinação do ponto de maior compressão, dos diâmetros e extensões de stent necessários, e ainda estabelecer a melhor via de acesso para o tratamento. Recentemente publicamos nossa sugestão de classificação de OVI, que contempla o local de maior compressão na região da confluência das veias ilíacas e o segmento venoso caudal livre de obstrução^30^ (Figuras 2 e 3).

**Figura 2:** Reconstrução multiplanar da angiotomografia venosa para a identificação do ponto de maior compressão ou obstrução.

**Figura 3:** Cálculo do grau de obstrução presente no ponto de maior compressão em membros portadores de insuficiência venosa crônica com classificação clínica, etiológica, anatômica e patofisiológica (CEAP) C3-6.

Pouquíssimos estudos mostraram a capacidade da angiorressonância no diagnóstico dessas obstruções, e apenas um estudo comparou o método com o IVUS^31^. Massenburg et al.^31^ compararam retrospectivamente a ressonância nuclear magnética com o IVUS em 46 pacientes portadores de sintomas clínicos sugestivos de OVI. Os autores verificaram sensibilidade de 100%, especificidade de 22,7%, valor preditivo positivo de 58,5%, e valor preditivo negativo de 100%.

Estudos demonstraram a superioridade do IVUS em relação à venografia no diagnóstico da OVI^22^ ^,^ ^24^ ^,^ ^29^ ^,^ ^32^. O método, além de determinar o grau mecânico da compressão, é o único capaz de verificar com precisão a presença de obstruções intraluminais (traves, trabéculas e membranas), as características da parede, a presença de trombos residuais e, sobretudo, possibilita a definição precisa do local e do grau de redução da área de secção transversal do vaso. A venografia, além de subestimar o grau de obstrução em aproximadamente 30% dos casos, pode falhar em identificar obstrução acima de 50% em até 25% dos casos^15^ ^,^ ^32^ ^,^ ^33^.

Na atualidade, o IVUS é considerado o padrão-ouro no diagnóstico da OVI. Alguns estudos indicam sensibilidade de 90% no diagnóstico dessas obstruções, sobretudo em pacientes portadores de IVC avançada. Ele é fundamental para confirmar e documentar do grau da obstrução, para determinar o segmento a ser recoberto com o stent (influxo e efluxo adequados) e, sobretudo, para verificar a ocorrência de obstrução residual e o sucesso do tratamento^29^ ^,^ ^32^ ^,^ ^33^ (Figura 4).

**Figura 4:** Venografia e ultrassom intravascular na determinação do grau e segmento obstruído: (A) Membro Direito; (B) Venografia; (C) Membro esquerdo; * Confluência das veias ilíacas interna e externa; b- Veia Ilíaca comum; c- Confluência das veias ilíacas externa e interna.

## TÉCNICA DE TRATAMENTO

Para o tratamento endovascular, o uso de subtração digital, colimação e a técnica de *road-map* são importantes para diminuir o tempo de exposição à radiação do paciente e dos colaboradores presentes na sala. Deve-se lembrar que muitos pacientes estão em idade fértil, e todas as medidas de radioproteção devem ser observadas^34^. Na maioria dos casos, o procedimento é realizado apenas com o uso de anestesia local e sedação, mas, nos casos de acesso poplíteo e jugular, em que o procedimento pode ser desconfortável, e de OPTVI, em que o tratamento do segmento ocluído pode ser doloroso e demorado, pode ser necessária sedação profunda ou anestesia geral. Recomenda-se a solicitação de perfil hormonal e de teste sorológico de gravidez ou espermograma para os pacientes em idade fértil.

Nossa preferência é a realização de acesso retrógrado na veia femoral, no terço médio da coxa ipsilateral à obstrução, guiado por ultrassom. Esse acesso permite uma abordagem mais curta e direta ao local da obstrução, permitindo o uso de cateteres e guias mais curtos, o que confere maior poder de torque e manobrabilidade. Abordagens alternativas nos casos de oclusão da veia femoral são: veia femoral profunda, veia jugular interna e veia poplítea. O acesso na virilha na maioria das vezes não é apropriado, porque muito frequentemente é necessário o prolongamento do stent abaixo do ligamento inguinal. Embora a pressão existente no interior das artérias limitar o uso de bainhas maiores do que 6 ou 7F, não existe qualquer restrição no sistema venoso. A bainha 11F é a que mais frequentemente utilizamos.

A venografia é o primeiro método de imagem a ser utilizado. Verificamos inicialmente a adequação do influxo, para determinar o ponto em que devemos posicionar a extremidade inferior do stent. Ele deve evitar o aprisionamento do orifício da veia femoral profunda, mas, em alguns casos, pode ser necessária até mesmo a invasão dessa veia para garantir um influxo adequado^30^. A venografia é útil na identificação da extensão do segmento obstruído. Entretanto, nem sempre isso é possível e, por esse motivo, deve-se sempre realizar projeções multiplanares e investigar a presença de sinais indiretos, como a presença de colaterais. A técnica de venografia anterógrada, com auxílio de manobra de Valsalva ou insuflação de balão, às vezes é necessária. A venografia é útil quando positiva, mas, quando negativa, é imprescindível a utilização do IVUS, para confirmar a ausência de compressões e verificar a presença de lesões intraluminais (traves, membranas e trombos residuais).

Na maioria das vezes, a ultrapassagem das obstruções é feita sem dificuldade, com cateteres e guias hidrofílicos. Em oclusões longas, a ultrapassagem da lesão pode ser bastante trabalhosa e exigir múltiplas projeções, conhecimento da anatomia e utilização de diversas pontas e perfis de cateteres. No sistema venoso, não é incomum a presença de alterações anatômicas. Sempre realizamos acesso venoso ecoguiado e venografia bilateral. Em todas as situações, sobretudo nas oclusões longas, quando existe uma rica rede de colaterais,, é fundamental a injeção de meio de contraste no interior da veia cava inferior (VCI) após a ultrapassagem do segmento obstruído, para confirmar o posicionamento do cateter no interior desse vaso. Por segurança, sempre estacionamos o fio-guia, com o auxílio do cateter na veia subclávia.

Após a venografia, o cateter de IVUS é introduzido sobre o fio-guia através da bainha. Com a utilização do *software* padrão, os diâmetros e a área do lúmen venoso de cada segmento devem ser verificados [VCI, veia ilíaca comum (VIC), veia ilíaca externa (VIE) e veia femoral]. A importância do IVUS pode ser salientada pela sua capacidade de verificar a presença de obstruções difusas, membranas e traves intraluminais, que podem se prolongar além do ponto de maior compressão, sobretudo na confluência das veias ilíacas.

Em nossa casuística, em 20 a 30% dos casos de síndrome pós-trombótica (SPT) existe a associação entre obstruções focais e difusas. No sistema arterial, o grau de obstrução é calculado comparando-se a lesão focal com o lúmen arterial adjacente normal. Isto é aceitável porque as lesões difusas são raras nas artérias. Mas, nas veias, lesões difusas são tão comuns, sobretudo nas obstruções pós-trombóticas, que a aplicação desse método pode levar a uma subestimação do grau de obstrução. No sistema venoso, deve-se calcular o grau de obstrução utilizando o valor da área anatômica considerada normal para o segmento (VIC: 200 mm^2^; VIE: 150 mm^2^; veia femoral comum: 125 mm^2^)^35^. Esses valores são necessários para garantir que haja escoamento e, consequentemente, pressão venosa periférica normal.

Deve-se lembrar que, embora a acurácia do IVUS seja superior à da venografia, ele pode falhar, particularmente na confluência das veias ilíacas. Nesse local, o cateter do IVUS pode ficar em posição não coaxial ao eixo do vaso, e o lúmen no quadrante oposto aparecerá alongado e obscuro, além da distância focal da sonda. Se houver dúvida na análise das imagens, pode-se usar a insuflação parcial do balão de angioplastia, com baixa pressão, com o objetivo de verificar se existem pontos de acinturamento, ou recorrer às imagens da TC pré-operatória para verificar o ponto exato onde ocorre a maior compressão^30^.

Durante a passagem do cateter de IVUS, deve ser verificada a presença, o grau e a extensão da obstrução, e marcados os diâmetros e as áreas. Ao realizar essas medidas, o operador deve determinar o local em que a extremidade inferior do stent deve ser posicionada, escolhendo um marco ósseo anatômico de referência, observado à fluoroscopia, que esteja abaixo da obstrução mais caudal. O operador deve também marcar o local exato da confluência das veias ilíacas, vista ao IVUS, e o local onde há a maior compressão, e a possível presença de estruturas intraluminais obstrutivas além desse ponto. Dessa forma, a fluoroscopia deve ser usada para determinar onde a extremidade superior e inferior do stent devem ser posicionadas. A borda proximal do stent pode situar-se na parte superior, média ou inferior das vértebras lombares a partir da borda inferior da L3 até a borda inferior de L5. Em casos pós-trombóticos, pode ser tão alta como a borda inferior de L2.

O cateter de IVUS é removido e um cateter balão de angioplastia é usado para dilatar todo o segmento venoso obstruído. Durante essa etapa, de pré-dilatação, geralmente usamos balões convencionais, tipo XXL® (4 -5 ATM) (Boston Scientific, Natick, MA, EUA) de 16 mm, ou de 18 mm para os casos de SPT. Nas obstruções pós-trombóticas, oclusões e nas lesões presentes em pontos de compressão e confluências venosas, pode ocorrer maior resistência à dilatação. Nessa situação, pode ser necessário o uso de balões de baixa complacência e alta pressão, tipo Atlas Gold® (6 -18 ATM) (Bard, Tempe, EUA). Em algumas situações, a pré-dilatação com balões de menor perfil, baixa complacência e alta pressão também pode ser necessária, para que seja possível avançar os cateteres balões de maior perfil.

O cateter balão é então removido e os stents são inseridos para que seja coberta toda a área previamente dilatada, impedindo que ocorra recolhimento elástico. Múltiplos stents podem ser necessários para alcançar esse objetivo. Nossa preferência ainda são os stents de malha trançada em liga de cromo, cobalto, níquel e outros metais, denominado Elgiloy® (Wallstent®, Boston Scientific, Natick, EUA). Na maior parte dos trabalhos em que se verificaram a efetividade clínica e a segurança a longo prazo, foi utilizado esse tipo de stent^14^ ^,^ ^23^ ^,^ ^35^ ^,^ ^36^. Estudos recentes vêm verificando a aplicabilidade clínica dos stents venosos dedicados de nitinol, demonstrando resultados iniciais bastante promissores; entretanto, ainda não temos estudos conclusivos e acompanhamento de perviedade a longo prazo^37^ ^-^ ^40^.

É recomendável recobrir todo o segmento obstruído, considerando-se as medidas obtidas pela angiotomografia e/ou pelo IVUS previamente realizados. As taxas de oclusão não estão relacionadas com a extensão ou com a quantidade de stents inseridos, mas sim com a cobertura incompleta do segmento de obstrução, que pode determinar influxo ou efluxo inadequado. Prefere-se, em geral, stents com diâmetro 2 mm maior do que aquele usado no balão de pré-dilatação. Os stents mais utilizados são os de diâmetro de 16, 18 e 20 mm. O stent de malha trançada diminui em comprimento quando pós-dilatado com o cateter balão; dessa forma, deve-se ter cuidado para garantir que ele acabe no local de ancoragem escolhido. Quando é necessário o uso de mais do que um stent, é importante que haja uma sobreposição de ao menos 3 a 5 cm, pelo mesmo motivo. A ocorrência de angulações aumenta a probabilidade de reobstrução em regiões de curvas acentuadas; nesses pontos, a sobreposição necessária pode ser ainda maior.

Na atualidade, existe controvérsia em relação ao comprimento do stent que deve invadir a VCI^41^ ^,^ ^42^. Na técnica original descrita por Raju & Neglén^12^ ^,^ ^13^ ^,^ ^21^ ^,^ ^23^, e ainda hoje utilizada pela grande maioria dos autores, o Wallstent® é posicionado ao menos 2 a 3 cm no interior desse vaso. Segundo esses mesmos autores, quando essa conduta não é observada, ocorre reobstrução proximal em 40% dos casos, por esmagamento e migração distal do stent^15^ ^,^ ^43^ ^,^ ^44^. Essa técnica é fundamental, porque o ponto de maior compressão, em mais da metade dos casos, ocorre na confluência ou acima dela^45^ ^-^ ^47^. Em nossa casuística, verificamos que a obstrução ocorre abaixo da confluência das veias ilíacas em 41,6% dos casos, ao nível da confluência em 34,5%, e acima dela, já dentro da VCI, em 23,9%^30^.

Na técnica de inserção do Wallstent® no interior da cava, pode ocorrer o aprisionamento e trombose venosa da veia ilíaca contralateral. Estudos clínicos e metanálises verificaram uma prevalência de TVP contralateral em 1,1 a 2,2% dos casos^14^ ^,^ ^23^. Em estudo randomizado^4^ e em nossa experiência clínica acumulada, com mais de 250 casos tratados, tivemos apenas dois casos de TVP em membro contralateral (1%), ambos em pacientes portadores de história clínica de TVP prévia e que não fizeram o uso recomendado do anticoagulante oral.

Recentemente, Murphy et al.^41^, em estudo retrospectivo, compararam a técnica utilizando o Wallstent® isolado com a inserção de Cook-Z-Stent® (Cook Medical, Bloomington, EUA) associado ao Wallstent® (Boston Scientific, Natick, EUA), técnica híbrida proposta por Raju et al.^48^. Verificou-se uma perviedade cumulativa contralateral de 99 e 90% nos grupos Z-Stent® e Wallstent®, respectivamente (p<0,001), após 5 anos de seguimento clínico. Entretanto, esse estudo apresentou algumas limitações importantes. O grupo submetido ao Z-Stent® foi comparado com uma série histórica de casos; assim sendo, a experiência adquirida pode ter influenciado a seleção de pacientes, as técnicas e os resultados, tornando os dois grupos não perfeitamente comparáveis.

Caliste et al.^49^ realizaram estudo retrospectivo que verificou a incidência de TVP ilíaca contralateral após o uso de stent através da confluência iliocaval. Estudaram 41 casos, 39 (95%) dos possuíam obstruções pós-trombóticas, e em 22 (54%) as obstruções envolviam a VCI. Quatro pacientes (9,7%) evoluíram com TVP contralateral, sendo que três desses pacientes estavam com anticoagulação inadequada. Apenas 2,4% pacientes que estavam anticoagulados adequadamente evoluíram com TVP contralateral (p = 0,0004).

Deve-se considerar que não é incomum a presença de compressão ou obstrução bilateral, sobretudo em pacientes portadores de SPT. Sabe-se, que a venografia e, algumas vezes, até mesmo o IVUS podem falhar na identificação de obstruções concomitantes, sobretudo nos pontos de confluência, p que pode limitar a presença de influxo adequado e aumentar a possibilidade de trombose venosa. Por esse motivo, preconiza-se o estudo multiplanar das imagens angiotomográficas no período pré-operatório, corrigindo os ângulos dos vasos estudados, para que possam ser verificados os pontos de compressão do eixo ilíaco-cavo bilateral, que devem ser confirmados com o IVUS intraoperatório. Em nosso meio, não há disponibilidade do Z-Stent, e, dessa forma, permanecemos utilizando a técnica original proposta por Raju et al^12^ ^,^ ^13^ ^,^ ^21^ ^,^ ^23^. Nos casos em que existe obstrução bilateral acima de 50% (confirmada pelo IVUS) e/ou presença de compressão em VCI, o que ocorreu em 11% dos casos, em nossa casuística, optamos pela técnica de stents em paralelo (*double barrel*), técnica inicialmente proposta por Neglén et al.^47^ (Figura 5).

**Figura 5:** Angioplastia com colocação de stent bilateral em paciente com classificação clínica etiológica, anatômica e patofisiológica (CEAP) C6, portador de obstrução severa comprometendo veias ilíacas bilaterais: (A) Úlcera varicosa unilateral (insuficiência venosa crônica CEAP C6S); Presença de compressão severa bilateral de ilíacas: (B, C) Angiotomografia; (D) ultrassom intravascular, confirmando obstrução severa bilateral (>50%); (E) Resultado satisfatório com implementação de stent pela técnica de *double-barrel*.

O cateter balão é então reintroduzido para a pós-dilatação do stent. Usamos os mesmos balões de 16 mm como padrão e balões de 18 mm para casos de SPT, com muito cuidado no avanço e recuo destes, o que deve ser realizado com o balão totalmente desinsuflado, introduzido em movimento giratório, e sem resistência, para que não haja perfuração de sua malha e deslocamento do stent. Deve sempre ser verificado se existem lesões craniais ou caudais às bordas do stent e, sobretudo, a área final do lúmen venoso, que deve se aproximar dos parâmetros anatômicos mínimos previamente descritos, sendo que o IVUS é o único método que permite fazer isso com precisão^50^ ^,^ ^51^. O lúmen deve ter forma regular, e o stent deve ter boa aposição à parede venosa. Às vezes, em casos de SPT de difícil dilatação, pode ser necessário o uso de balões de alta pressão. Deve-se lembrar que estudos experimentais indicam que obstruções residuais de 20% podem ser o suficiente para manter a ectasia e os sintomas, levando à falha clínica do tratamento^51^. Em nossa casuística, em 30% dos casos a venografia falhou no diagnóstico da obstrução residual quando comparada ao IVUS, demonstrando a importância desse último método para o sucesso do tratamento (Figura 6).

**Figura 6:** Ultrassom intravascular (*intravascular ultrasound*, IVUS) de controle final. Em (A), flebografia inicial demonstrando sinais diretos e indiretos de obstrução de veia ilíaca esquerda, confirmada pelo IVUS (área no ponto de maior compressão de 53,5 mm^2^). Em (B), flebografia de controle aparentemente demonstrando resultado satisfatório, não confirmado pelo IVUS (área no ponto de maior compressão de 54,1 mm^2^). Em (C), resultado final após angioplastia com balão de baixa complacência e alta pressão.

A venografia é o método final de avaliação do resultado. O canal do segmento tratado deve estar livre de irregularidades ou obstruções. As colaterais devem desaparecer, porque o canal principal de escoamento do fluxo é a via de menor resistência; entretanto, quando elas estão presentes há muito tempo e são muito dilatadas, não é incomum que, na venografia final, elas ainda sejam parcialmente visíveis, mas o fluxo deve ser maior e preferencial pelo canal principal, e as contralaterais não devem conter represamento do fluxo. Após a retirada da bainha introdutora, é aplicada compressão manual no local da punção.

## USO DE ANTITROMBÓTICOS

No pré-operatório da SMT ou da ONTVI, administramos ácido acetilsalicílico (AAS) 100 mg/d e clopidogrel 75 mg/d, com início 7 dias antes da realização do procedimento. Para os pacientes portadores de OPTVI ou SC que não estejam mais fazendo uso de anticoagulantes orais, aplica-se a mesma conduta. Naqueles que estão fazendo uso de anticoagulantes, deve-se solicitar a suspensão do seu uso no período necessário, para que ocorra a suspensão de seus efeitos; no caso do uso de varfarina, visando a uma razão normalizada internacional (*international normalized ratio*, INR) entre 1,5-2,0 no dia da angioplastia. No intraoperatório, os pacientes recebem no mínimo 5.000 U de heparina não fracionada (100 U/kg). Durante a internação, permanecem em anticoagulação plena com heparina de baixo peso molecular.

Após a alta hospitalar, que na maioria dos casos ocorre no mesmo dia ou na manhã seguinte nos casos de SMT/ONTVI, quando é considerado sucesso técnico, preconiza-se o uso de clopidogrel 75 mg/d associado ao AAS 100 mg/d por 6 meses, e AAS 100 mg/d para o resto da vida. Nos pacientes portadores de SC/OPTVI e recanalização de oclusões longas, é indicado o uso de anticoagulação com anti-vitamina K e controle rigoroso de INR, ou com os novos anticoagulantes orais diretos, por um período mínimo de 6 meses. Os pacientes que já faziam uso no pré-operatório (trombofilias e tromboses de repetição) continuam anticoagulados por tempo indeterminado no pós-operatório. Em nossa prática, esse tipo de protocolo tem sido associado a boa resposta terapêutica e perviedade a longo prazo; entretanto, devemos lembrar que ainda não existem estudos que tenham verificado qual o melhor regime de tratamento antitrombótico nesse grupo de pacientes^4^.

## SEGUIMENTO PÓS-OPERATÓRIO

Como já mencionado anteriormente, na visita inicial verificamos e anotamos a intensidade da dor pela EVAD, a classificação clínica CEAP e o VCSS, além de submetemos os pacientes ao questionário de qualidade de vida SF-36 e anotarmos os diâmetros dos membros no meio da coxa, perna e mediopé. Esses dados também são verificados nas visitas pós-operatórias, que são realizadas, rotineiramente, com 1, 3, 6 e 12 meses, e anualmente a partir de então. Nessas visitas, o paciente é submetido também a eco-Doppler venoso colorido e são verificados os mesmos parâmetros acima expostos^7^. Nas visitas de 6 meses e 1 ano, solicitamos também raio X de pelve para verificar a integridade e o posicionamento do stent. Nos pacientes assintomáticos, caso haja suspeita de obstrução intra-stent ao eco-Doppler, ou haja recidiva dos sintomas, o paciente é submetido a nova angiotomografia e venografia com passagem de cateter de IVUS, com possível dilatação do ponto da obstrução com cateter balão que tenha o mesmo diâmetro do stent. Em alguns casos, pode ser necessária a colocação de stent proximal ou distal ao segmento tratado.

## RESULTADOS

Vários estudos retrospectivos, metanálises e diretrizes de sociedades sugerem que o implante de stent ilíaco é eficaz, seguro, e deve ser considerado o tratamento de escolha para os pacientes portadores de obstruções ilíacas, associadas à presença de refluxo ou não, e para portadores de sintomas graves e classificação CEAP avançada^14^ ^,^ ^23^ ^,^ ^36^ ^,^ ^52^ ^,^ ^53^.

Em recente estudo randomizado, duplo-cego, comparativo entre tratamento clínico e endovascular em pacientes portadores de obstrução venosa ilíaca e classificação CEAP avançada, verificamos que 28% dos pacientes em acompanhamento em ambulatório de insuficiência venosa preenchiam os critérios de inclusão do estudo, e que 60% dos membros estudados possuíam obstrução ilíaca ≥50% ao IVUS. Isso demonstra a alta prevalência dessa situação clínica em um hospital terciário especializado, considerando que, nesse protocolo, apenas os pacientes com CEAP C3-6 e que estavam em acompanhamento por mais de 1 ano sem resposta terapêutica foram investigados. O sucesso técnico imediato foi de 100% e não houve complicações graves no período perioperatório. Após 6 meses de acompanhamento, a média do escore da EVAD diminuiu de uma mediana de 8 para 2,5 em pacientes que receberam stents, e de 8 para 7 em pacientes que estavam recebendo apenas tratamento clínico (p < 0,001). O VCSS caiu de uma mediana de 18,5 para 11 no grupo tratado com stent, e de 15 para 14 no grupo de tratamento clínico (p < 0,001). O questionário de qualidade de vida SF-36 passou de um escore mediano total de 53,9 para 85,0 com implante de stent, e de 48,3 para 59,8 após tratamento clínico (p < 0,001). Não houve fratura ou migração do stent, e as taxas de perviedade primária, primária assistida e secundária foram de 92%, 96% e 100%, respectivamente (mediana: 11,8 meses; intervalo: 6-18 meses)^4^.

Existem poucos estudos que tenham verificado os desfechos clínicos exclusivamente em portadores de SMT ou ONTVI. A maioria dos estudos mistura obstruções trombóticas e não trombóticas, e muitos incluem o tratamento do refluxo venoso superficial. Aparentemente, esse grupo de pacientes apresenta desfecho clínico favorável, particularmente em termos de alívio da dor e cura de úlceras venosas.

Raju e Neglén^54^ observaram que, após 2,5 anos da colocação do stent, houve alívio completo da dor, alívio completo do inchaço e cicatrização sustentada de úlceras em 77%, 53% e 76%, respectivamente. Em estudo posterior, verificaram índice de permanência de cicatrização de ferida de 62% em 5 anos de acompanhamento clínico^53^. Ye et al.^55^ mostraram taxas de alívio da dor, do inchaço e de cicatrização da úlcera em 87%, 88% e 74%, respectivamente, em 101 membros, com acompanhamento médio de 4 anos. Os escores de qualidade de vida melhoraram significativamente em ambos os estudos. Raju e Neglén^54^ mostraram resultado semelhantes em subgrupos de membros portadores de ONTVI isolada e associada a refluxo, mesmo que este permanecesse sem tratamento.

Outro estudo interessante foi o de Meng et al.^56^, o qual verificou que, na presença de obstrução ilíaca, só 13% dos membros portadores de varizes operadas tiveram alívio significativo de sintomas após 2 meses de acompanhamento. Embora a perviedade cumulativa seja menor nos pacientes portadores de OPTVI, os resultados ainda assim são bastante satisfatórios. Neglen et al. verificaram perviedade primária, primária assistida e secundária de 57%, 80% e 86%, respectivamente, em 5 anos de acompanhamento^53^. Estudos apontam que a perviedade secundária, com uma média de acompanhamento de 4 a 7 anos, seja entre 74% a 89%^23^. Pacientes tratados por oclusão crônica possuem índices de recanalização de 83% a 95%^14^ ^,^ ^23^. Raju e Néglen^57^ verificaram, em 139 membros tratados por oclusão crônica, uma perviedade secundária de 66% aos 4 anos de acompanhamento, e taxas de alívio da dor e edema de 79% e 66%, respectivamente. No estudo que relatou recanalizações de obstruções totais e longas no segmento fêmoro-ilíaco-cavo, a perviedade secundária foi de 66% a 89%, com um acompanhamento de 4 a 7 anos^23^. A cicatrização da úlcera em pacientes pós-trombóticos é menor do que na coorte não trombótica; entretanto, mesmo nesse grupo, foi demonstrada uma taxa cumulativa de cicatrização de ferida de 60% em 5 anos^58^. Em pacientes com úlceras que não cicatrizam, deve-se investigar a presença de obstrução intra-stent, refluxo no sistema venoso superficial e pérfuro-comunicante.

## CONCLUSÃO

A obstrução venosa ilíaca é altamente prevalente, sobretudo nos pacientes portadores de IVC avançada. Apesar de poder ser assintomática, frequentemente é associada a sintomas incapacitantes e importante piora da qualidade de vida. Sua presença ainda é pouco investigada, e a falta de critérios bem estabelecidos para seu diagnóstico não invasivo contribui para que muitos dos pacientes não recebam o diagnóstico e tratamento adequados. O tratamento endovascular hoje é considerado o padrão-ouro. Ele pode ser realizado com altas taxas de sucesso técnico, baixos índices de morbimortalidade, altos índice de perviedade e sucesso terapêutico, desde que sejam consideradas as características anatômicas e fisiopatológicas que ocorrem na presença dessas obstruções.
